# A multicenter retrospective study of heterogeneous tissue aggregates obstructing ventricular catheters explanted from patients with hydrocephalus

**DOI:** 10.1186/s12987-021-00262-3

**Published:** 2021-07-21

**Authors:** Prashant Hariharan, Jeffrey Sondheimer, Alexandra Petroj, Jacob Gluski, Andrew Jea, William E. Whitehead, Sandeep Sood, Steven D. Ham, Brandon G. Rocque, Neena I. Marupudi, James P. McAllister, David Limbrick, Marc R. Del Bigio, Carolyn A. Harris

**Affiliations:** 1 Wayne State University Dept. of Biomedical Engineering, 6135 Woodward Avenue, Detroit, MI 48202 USA; 2 Wayne State University Dept. of Chemical Engineering and Materials Science, 6135 Woodward Avenue, Detroit, MI 48202 USA; 3 Dept. of Neurosurgery, Wayne State University School of Medicine, 540 E. Canfield Avenue, Detroit, MI 48201 USA; 4 Riley Hospital for Children at IU Health, 705 Riley Hospital Drive, Indianapolis, IN 46202 USA; 5 Texas Children’s, 6701 Fannin Street, Suite 1230.01, Houston, TX USA; 6 Departments of Neurosurgery and Pediatric Neurosurgery, Wayne State University School of Medicine and Children’s Hospital of Michigan, 3901 Beaubien Boulevard, 2nd Floor Carl’s Building, Detroit, MI 48201 USA; 7 Department of Neurosurgery, University of Alabama At Birmingham, Birmingham, AL USA; 8 Children’s Hospital of Michigan Dept. of Neurosurgery, 3901 Beaubien Boulevard, 2nd Floor Carl’s Building, Detroit, MI 48201 USA; 9 School of Medicine Dept. of Neurological Surgery, Washington University, 425 S. Euclid Avenue, St. Louis, MO 63110 USA; 10 School of Medicine Dept. of Neurological Surgery, Washington University, 660 S. Euclid Avenue, St. Louis, MO 6311 USA; 11 Department of Pathology, Max Rady College of Medicine, Rady Faculty of Health Sciences, University of Manitoba, Winnipeg, MB Canada

**Keywords:** Hydrocephalus, Biobank, Ventriculoperitoneal shunt, Shunt failure, Shunt obstruction, Retrospective cohort, Multicenter, Surgical outcomes

## Abstract

**Background:**

Implantation of ventricular catheters (VCs) to drain cerebrospinal fluid (CSF) is a standard approach to treat hydrocephalus. VCs fail frequently due to tissue obstructing the lumen via the drainage holes. Mechanisms driving obstruction are poorly understood. This study aimed to characterize the histological features of VC obstructions and identify links to clinical factors.

**Methods:**

343 VCs with relevant clinical data were collected from five centers. Each hole on the VCs was classified by degree of tissue obstruction after macroscopic analysis. A subgroup of 54 samples was analyzed using immunofluorescent labelling, histology and immunohistochemistry.

**Results:**

61.5% of the 343 VCs analyzed had tissue aggregates occluding at least one hole (n = 211) however the vast majority of the holes (70%) showed no tissue aggregates. Mean age at which patients with occluded VCs had their first surgeries (3.25 yrs) was lower than in patients with non-occluded VCs (5.29 yrs, p < 0.02). Mean length of time of implantation of occluded VCs, 33.22 months was greater than for non-occluded VCs, 23.8 months (p = 0.02). Patients with myelomeningocele had a greater probability of having an occluded VC (p = 0.0426). VCs with occlusions had greater numbers of macrophages and astrocytes in comparison to non-occluded VCs (p < 0.01). Microglia comprised only 2–6% of the VC-obstructing tissue aggregates. Histologic analysis showed choroid plexus occlusion in 24%, vascularized glial tissue occlusion in 24%, prevalent lymphocytic inflammation in 29%, and foreign body giant cell reactions in 5% and no ependyma.

**Conclusion:**

Our data show that age of the first surgery and length of time a VC is implanted are factors that influence the degree of VC obstruction. The tissue aggregates obstructing VCs are composed predominantly of astrocytes and macrophages; microglia have a relatively small presence.

**Supplementary Information:**

The online version contains supplementary material available at 10.1186/s12987-021-00262-3.

## Background

Hydrocephalus is a condition that results in the abnormal enlargement of cerebral ventricles due to CSF accumulation caused by impaired CSF dynamics. The estimated incidence of hydrocephalus in the United States is 1 per 1100 people [1]. Hydrocephalus is currently treated surgically with either choroid plexus cauterization (CPC), endoscopic third ventriculostomy (ETV) or ventricular shunting; the latter is the most common. The ventricular shunt system, typically consisting of a distal catheter, a valve and a ventricular catheter, drains CSF from the ventricles and relieves intracranial pressure. However, these shunts have a failure rate of about 30% within a year of placement [2, 3], which rises to 85% after 15 years [4]. The estimated cost of hospital admissions due to shunt failures in the United States was estimated to be over $1 billion per annum in 2005 [5, 6].

Shunts fail for various reasons including proximal or distal catheter obstruction, valve failure, infection, distal catheter migration, shunt disconnection, over-drainage or a combination of these problems. Of these, obstruction of the proximal ventricular catheter is the main cause of failure in ventricular shunt systems in the pediatric age group [7]. A major cause of failure of VCs is the obstruction of these holes by tissue, restricting its drainage of CSF. Previous undertakings have sought to identify clinical risk factors and cellular mechanisms that could help predict and prevent ventricular catheter obstruction [8–17]. Factors such as age [18], number of surgeons involved in a surgery, etiology of hydrocephalus [19], and having a previous procedure before shunt placement [20] have been identified to be associated with greater odds of complications following implantation.

Although previous studies have identified cell types that are involved in the obstruction of the catheter holes [8, 13, 14], the mechanisms by which these cells cause the obstruction are not completely understood, therefore a topic of debate among researchers. Flow distribution through the holes of the proximal catheter [21], shear [22] and suction of tissue due to over drainage [23] have also been implicated as playing a role in proximal obstruction. We hypothesized that clinical observations would reliably predict the degree of VC obstruction and the composition of the obstructing tissue aggregates. To test this hypothesis and shed light on the mechanisms responsible for shunt failure, we set up a multi-center shunt biobank to study failed shunts [7]. Using this resource, we aim to build on the observation from previous studies and establish links between clinical data, biological reactions, and shunt outcomes. The long-term goals are to model, predict and engineer solutions to shunt obstruction.

## Methods

### Ethics approval and patient population

Consent and sample collection has been described in detail in our recently published work [7]. Written informed consent was obtained from all patients or their legally authorized representative and the study received approval from the Wayne State University IRB. Samples were collected from individuals with any hydrocephalus etiology except normal pressure hydrocephalus. Patients were evaluated by local centers according to their individual guidelines, and samples were only collected if the shunt malfunction indicated surgical intervention.

## Sample collection

Collection centers included Children’s Hospital of Michigan and Wayne State University (WSU) as the coordinating center, St. Louis Children’s Hospital and Washington University School of Medicine in St. Louis (WUSM), Texas Children’s Hospital -Baylor College of Medicine (TEX), Riley Children’s Hospital—Indiana University Health (RC), and the Children’s Hospital of Alabama at University of Alabama Birmingham (ALA).

After removal by a surgeon, VCs were placed in a solution of 4% (w/v) paraformaldehyde (PFA). Samples were de-identified before being shipped to the coordinating center at room temperature. Upon arrival, shunt components were moved to a solution of sterile filtered 1X PBS with 0.01% (w/v) sodium azide and stored at 4 °C. The solution was refreshed monthly.

Clinical variables including dates of surgery, hydrocephalus etiology, demographics, surgeon-specified cause for hardware removal, physician performing procedure, shunt configuration, infection status at revision surgery and use of electrocautery were collected and stored in REDCap (Research Electronic Data Capture: a secure, web-based software platform designed to support data capture for research studies). For a full list of all the variables collected please refer to our previously published work on our shunt biobank [7]. Mean length of time of implantation was calculated for each catheter by counting the number of days between the date of catheter insertion surgery and date of revision surgery.

Only samples placed intraventricularly—i.e. VCs and external ventricular drains (EVDs)—were included in analysis; other catheter types that would not routinely be exposed to intracranial pressure driven flow were excluded to reduce heterogeneity introduced by other medical comorbidities.

Surgeons at each of the five centers recorded a VC as having failed due to a ‘suspected proximal obstruction’ if the patient presented with one or more of the following symptoms: lethargy, headache, dizziness, nausea, vomiting, irritability, fever, or an altered level of consciousness and less commonly, increased seizure frequency, diplopia, weakness, or visual loss [24]. Before making a final decision on the necessity of a revision a surgeon may assess the patency of a shunt by performing a “shunt tap” or by using dilation of the ventricles proxy. It is important to note that although these symptoms are commonly used as indications of shunt malfunction by surgeons, there is variability in the degree to which a surgeon may rely on each individual symptom.

### Label-free explant imaging

Every hole of each catheter was photographed using a digital bright-field microscope. Images were captured by vertically positioning the equipment over each VC hole at a 5-cm distance with the microscope’s internal LED serving as the light source. AMCAP image capture software v-9.016 was used to capture images at 1.3-megapixel resolution and 40 × magnification. The investigator was blinded to sample identity. Each individual hole was binned into one of three categories based on the following rules:

If the hole showed no tissue aggregates or if it showed a scattering of cells or cellular debris it was classified as ‘N’ (no obstruction).
2) If the hole showed clear signs of cellular growth across the hole but this cellular growth was not occluding the entire opening of the hole, it was classified as a ‘G’ (growth).
If the hole was completely occluded by cellular growth sometimes presenting with a large tissue aggregate protruding out of the hole, it was classified as an ‘O’ (occluded).

### Ventricular catheter (VC) classification

Each catheter was classified overall depending on the number of holes meeting each classification using the following rules:

1. If one or more holes were designated as ‘O’—VCs with Occlusions.
2. If one or more holes designated as ‘G’- VCs with Growths.
3. If no tissue aggregates were visible—Empty VCs.

VC classifications were correlated with clinical parameters including patient age at surgery, patient weight, etiology, catheter type & shunt configuration, cause for shunt revision, whether the catheter was adherent to a ventricular wall or the CP, infection status at the time of revision, age of the patient at the time of their very first surgery. Measured parameters included number of cells/ holes, total cells/ catheter, obstructed volume/ catheter and number of inflammatory cells/ VC.

### Immunofluorescent (IF) labeling of explanted VCs

Explanted VCs were subjected to three rapid washes with phosphate-buffered saline and then incubated in 5 mg/ml of sodium borohydride (Sigma-Aldrich, Saint Louis, MO, USA) solution for 30 min at room temperature, 37 °C (RT). Next, the tissue on the VCs were permeabilized by incubating in 0.2% Triton X-100 (Sigma-Aldrich) solution for 30 min at RT. VCs were then incubated in BlockAid™ Blocking Solution (B10710, Life Technologies, Carlsbad, CA, USA) for 30 min at RT.

VCs were labeled with the following antibodies: GFAP (1:1000, PA1-10,004, Invitrogen), CD68 (1:100, MA5-13,324, Invitrogen Corp., Carlsbad, CA, USA), and TMEM-119 (1:1000, ab185333, AbCam, Cambridge, MA, USA). Samples were incubated with primary antibody for 24 h at RT. Subsequently samples were incubated with: Alexa Fluor 568 conjugated anti-chicken (1:200, ab175477), Alexa Fluor 405 conjugated anti-mouse (1:200, A-31553), and Alexa Fluor 660 conjugated goat anti-rabbit (1:200, A-21074), for 24 h at RT. Additionally, at the time of secondary antibody application, VCs were labelled with 1:1000 CyQuant (Sigma-Aldrich) for the identification of cell nuclei. Pilot experiments showed that CyQuant and antibodies easily penetrated 1 mm from the surface of a tissue sample.

After 24 h in secondary antibodies and CyQuant, the VCs were washed 4 times over 30 min with Hank's Balanced Salt Solution (HBSS) containing sodium azide. Last, the ventricular VCs were mounted onto a custom-built rotisserie holder which kept samples submerged under buffer solution while allowing us to easily rotate the sample to image all sides.

### 3D explant imaging

The samples in the biobank were divided into two broad groups: catheters from patients with 6 or more revisions and catheters from patients with less than 6 revisions. 30 samples were selected randomly from each of the two groups. 6 patients rescinded their consent; their samples were removed from the analysis. Resonance-scanning confocal microscopy was used to image the remaining 54 VCs (RS-G4 upright microscope, Caliber ID, Andover, MA, USA). Confocal images (1000 × 1000 × 800 μm) centered around each hole of each catheter were acquired using a Leica HC FLUOTAR L 25x/0.95 W VISIR objective (80 optical z-sections, 10 μm step size). Imaging was performed blinded to sample identity.

### Quantitative analysis

Data analysis from the confocal images was carried out blinded using Imaris 3D/4D Visualization & Analysis Software (Bitplane Inc., South Windsor, CT, USA). Once labelled and imaged, the raw z-stack images for each catheter hole were compiled using Imaris File Converter software. ‘Spots Detection’ function in Imaris was used to quantitatively assess the IF labels in each image. Cell type identification was carried out by counting colocalized signals from cytoplasmic IF labels with the nearest nuclei using a filter function. The ‘Batch’ feature in Imaris was used to process multiple images at once.

To obtain the count of CD68 positive immune cells excluding microglia, the total count of nuclei that colocalized for both TMEM119 and CD68 were subtracted from the total count of CD68 positive nuclei.

Volumes of obstruction for every hole of each VC were captured using the ‘Surface’ feature in Imaris. First quantification of the total volume of a hole was acquired by tracing the contour of the hole in each slice of a confocal z-stack. On the z-axis, the stack was initiated when the external surface of the VC was visible and terminated when the lumen was visible. The individual contours were then merged into a 3D contour surface. The ‘Surface’ feature was then used to render a 3D volume corresponding to the obstructing tissue by using the data from IF labels. The rendered 3D volume of obstructing tissue and the ‘total hole volume’ was then used to calculate percentage obstruction.

### Explanted tissue histopathology and immunohistochemistry (IHC)

VCs were photographed and examined using a dissecting microscope (up to 20 × magnification). Since conventional histology requires tissues that are large enough for microdissection, only ‘VCs with Growths’ and ‘VCs with Occlusions’ were prepared for analysis. VCs were cut in the cross-sectional plane to include only regions with visible tissue. Alternatively, tissues were extracted by making a longitudinal incision. The extracted tissues were dehydrated, embedded in paraffin, sectioned and mounted. Tissue sections were cut at 5 µm thickness and stained with hematoxylin and eosin (H&E). Based upon the predominant microscopic features, the tissues were broadly categorized by an experienced neuropathologist (MRD) into five groups: glial / brain tissue; choroid plexus (CP), inflammatory tissue, blood clot / debris. Additional histochemical stains included: Masson trichrome (to detect collagen), Perls’ Prussian blue (to detect hemosiderin), and Hucker-Conn Gram (to detect bacteria).

Immunohistochemistry (IHC) was performed using a Link 48 automated staining system (Agilent, Santa Clara, CA, USA) and primary antibody detection using the Dako EnVision labelled polymer system (Agilent). Primary antibodies are shown in Additional file 1: Table S1. Validations of all commercial antibodies are regularly performed in the College of American Pathologists (CAP)-accredited clinical pathology laboratory (Health Sciences Centre Winnipeg; Shared Health Manitoba). Cell types were identified based on morphologic and immunostaining criteria using a standard light microscope and representative images were photographed at 12.5 to 600 × magnification.

Sections were imaged on Leica TCS SP8 confocal laser scan microscope. Wholemounts were imaged using a 100 × /1.4 HC PL APO oil immersion objective lens, taken at scan zoom 1 (13,567.59 µm2 field area) or scan zoom 2 (3391.90 µm2 field area).

### Statistics

Data analysis was performed using GraphPad Prism v8.0 software. All data are presented as mean ± standard deviation (SD); n refers to the number of VCs. Shapiro–Wilk test was performed to examine if the data were normally distributed. All normally distributed data sets were analyzed using parametric tests (one-way ANOVA). Non-normal data sets were analyzed using a non-parametric test (Kruskal–Wallis). Categorical variables were analyzed using the Chi-Squared test. All P values below 5% (P < 0.05) were considered statistically significant; P < 0.05 (*), P < 0.01 (**), P < 0.001 (***) and P < 0.0001 (****).

Additionally, to estimate the fixed and random effects, the statistical software Stata 16.1 was used to fit a series of linear mixed models with a random intercept. A set of 7 covariates were simultaneously entered into the models to predict the following outcome variables: CyQuant, Cd68, GFAP, and TMEM119. The covariates (predictors) were age, sex, race, implant duration, number of revisions, surgery reason, and shunt type. Cell count data from the IF labelled samples imaged using confocal microscopy were used for this analysis (n = 54).

## Results

### Sample accrual and clinical data

A total of 343 explanted VCs were collected from 265 patients during revision surgeries performed between 5/15/2015 and 7/31/2020. Age at the time of revision surgery ranged between 36 days and 42.6 years, with a mean of 9.4 years and median of 8 years (SD = 8.01). 10.5% of the samples came from adults while 89.5% came from pediatric patients. The three most common catheter configurations were the 8-hole 3-side catheter (42.27%, n = 145), followed by 5-hole 4-side, (32.36%, n = 111) and 8-hole 4-side, 16% (n = 55). The less common catheter configurations included 4 side 4 holes (n = 25), 2 side 4 holes (n = 5), 2 side 5 holes (n = 1) and 4 side 10 holes (n = 1). 7.8% (n = 27) of the samples collected were external ventricular drain catheters (EVDs). Of these 27 EVDs, 9 were imaged using confocal microscopy. 8.7% (n = 30) of samples had positive CSF cultures during admission indicating infection. Intraluminal electrocautery (Bugbee wire monopolar electrocautery) was used on 12% (n = 41) of the VCs of which 14 were imaged using confocal microscopy. Bipolar electrocautery was used during removal of 30% (n = 103) of the VCs. A summary table of patient demographics, hydrocephalus etiologies, suspected causes of shunt malfunction for the 256 patients included in this study can be found in the Additional file (Additional file 1: Table S2). For a more thorough center-specific description of the samples, refer to our previous publication [7].

### Brightfield imaging and categorization of VC holes based on degree of obstruction

**Fig. 1:**
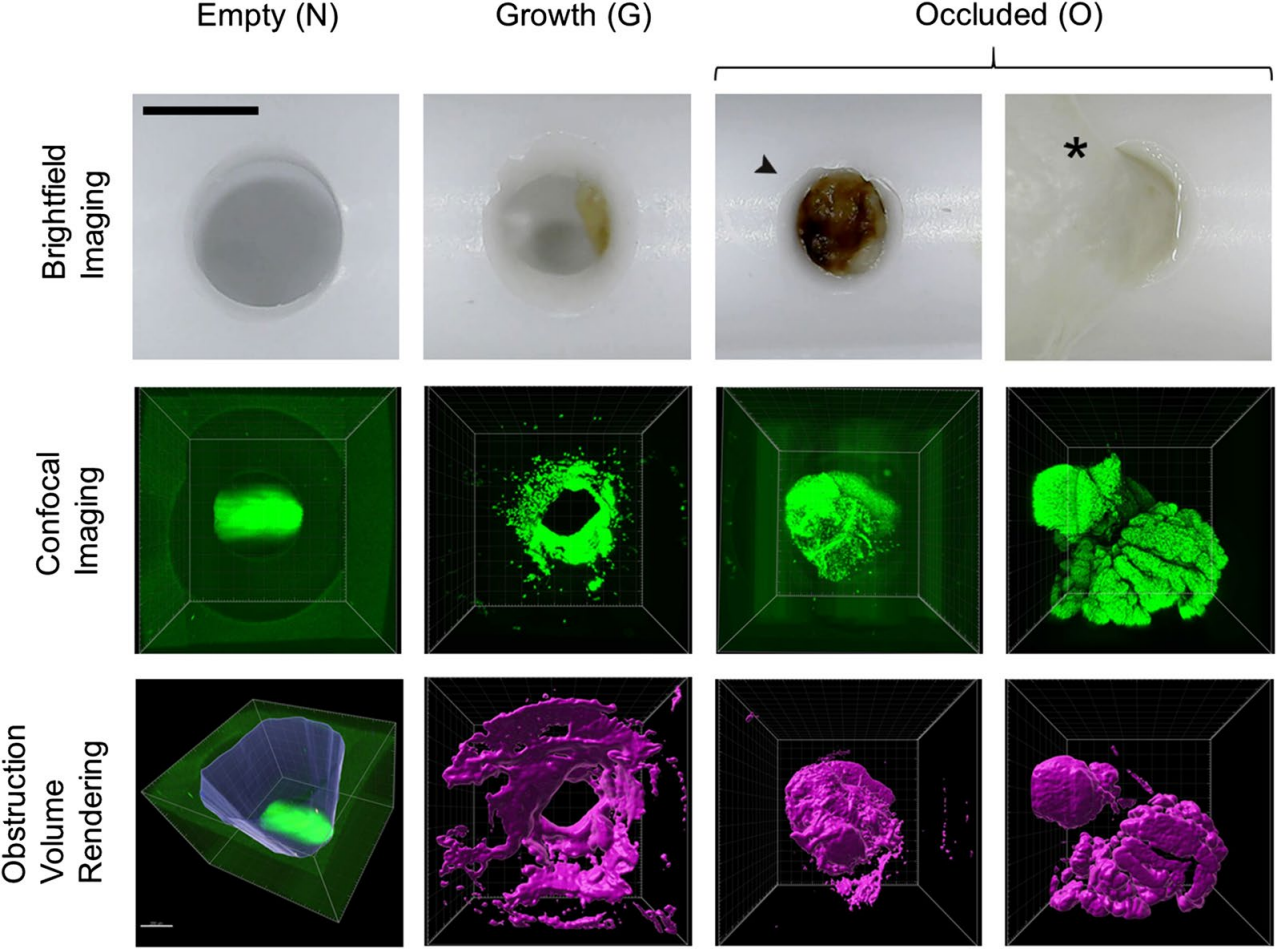
Representative images of individual holes in explanted ventricular catheters (VCs) showing each obstruction category. The first row shows brightfield images. The second row shows CyQuant fluorescent (green) labeling of nuclei. The third row shows volume rendering of the immunofluorescent labeling. A thin layer of cells is adherent to the VC surface in the G column. Two sets of images, under the ‘O’ hole category demonstrate two types of occluded holes. The first O column shows tissue filling the lumen of the VC at the base of the hole. The second O column shows tissue protruding out of the hole. (asterisk) The green area in the N column is due to an artefact in the trough of the VC lumen. Scale bar top left: 1000um

**Fig. 2:**
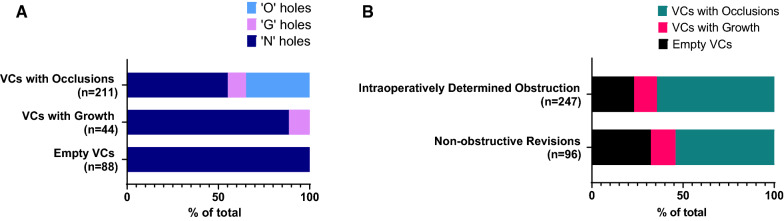
**A** Distribution of tissue in holes by ventricular catheter (VC) group. In VCs with Growths, 88% of holes were empty and 11% of holes showed Growths. In VCs with occlusions, 34% of holes were occluded, 10% had non-occluding growths, and 55% were empty. **B** Bar graphs showing VC categorization within the broad clustering of clinical obstructions versus non-obstructive revisions. Of those VCs that were determined intraoperatively to be obstructed, 23% were designated as ‘Empty VCs’, 13% as ‘VCs with Growths’ and 64% as ‘VCs with Occlusions’. Among VCs that were replaced for causes other than obstruction, 32% were designated as ‘Empty VCs’, 14% as ‘VCs with Growths’ and 54% as ‘VCs with Occlusions’

A total of 7946 holes from 343 VCs were evaluated. Depending on where the brain tissue separated from the tissue obstructing a hole during VC removal, tissue obstructions could be confined to a single hole or be contiguous with tissue masses stretching externally across multiple holes or in the lumen of the VC (O category—22.3%). Some holes exhibited significant cellular aggregation on the VC surface without complete obstruction (G category—7.6%). (Fig. 1) 70% of holes showed no tissue (N category). Considering the VCs as a whole, 61.5% (n = 211) had one or more holes classified as ‘O’, 12.8% (n = 44) had holes classified as ‘G’s but none as ‘O’, and 25.7% (n = 88) of VC were empty. (Fig. 2A) Chi-squared test showed that the proportion of samples that were verified to have occlusions were not significantly different between VCs that were determined intraoperatively to be obstructed.

### Relating VC obstruction classes to clinical parameters

**Fig. 3:**
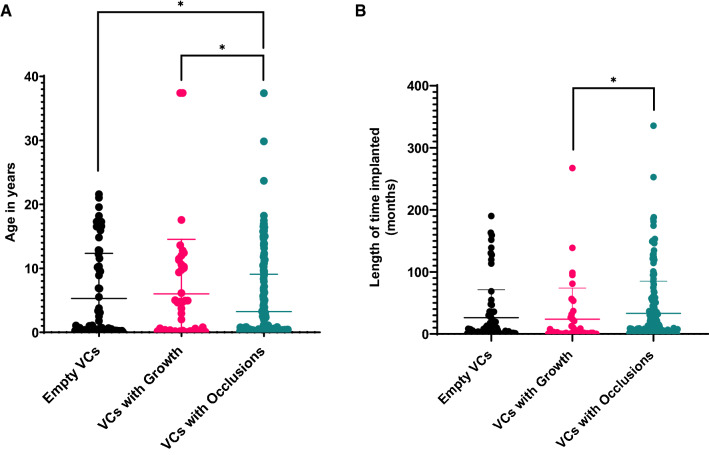
**A** Distribution of patient age at the time of first surgery across the three ventricular catheter (VC) obstruction classes. **B** Distribution of the length of time for which the patients have had the VC placed in their ventricular space before revision. Horizontal line indicates arithmetic mean, and error bars show one SD standard deviation above mean. Differences between groups were assessed using Kruskal–Wallis’s test with Dunn’s multiple comparisons test, *P < 0.05

The mean age at which the patient had their first surgery was significantly lower in patients whose explanted VCs showed occlusions than either the ‘Empty VCs’ (p = 0.0104) or the ‘VCs with Growths’ (p = 0.0245, Fig. 3A). The duration that the catheter was implanted before revision was also significantly longer in ‘VCs with Occlusions’ than in ‘VCs with Growths’ (p = 0.02) (Fig. 3B). A correlation was observed between patients with 8-side 4-hole VCs and lower obstruction by tissue aggregates (‘VCs with Occlusions’), however this relationship was not statistically significant (Chi square p = 0.07). Finally, we observed a significant difference between the frequency of VC obstruction categories among the hydrocephalus etiology: myelomeningocele (Chi square p = 0.0426). No significant differences were observed between the VC obstruction classes when comparing patient weight (p = 0.3622), number of prior revisions (p = 0.638), and age of the patient at the time of surgery (p = 0.2285). Of the 30 VCs that had positive CSF cultures during admission 17 were classified as ‘VCs with Occlusions’, 7 as ‘VCs with Growths’ and 5 as ‘Empty VCs’. Chi-squared test showed that infection status at the time of revision affected the proportion of occluded and empty VCs significantly (p = 0.26).

### Quantitative 3-D analysis after immunofluorescence labeling

**Fig. 4:**
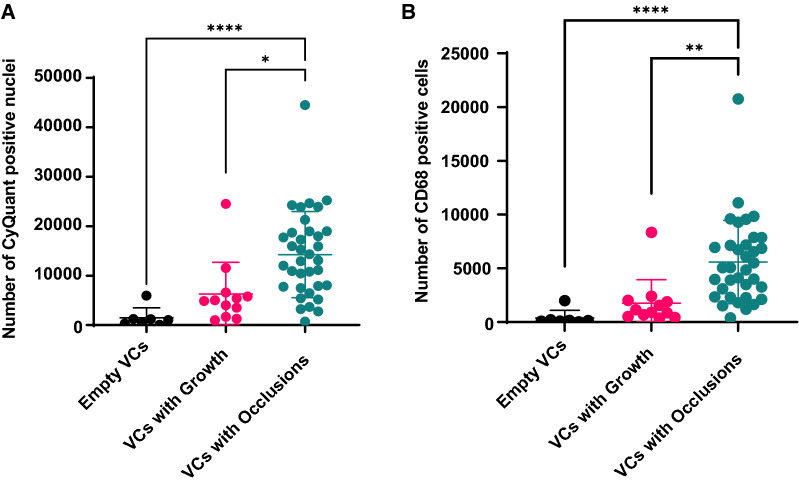
**A** Total number of cells (CyQuant positive nuclei) in each ventricular catheter (VC) obstruction-class. **B** Number of the average number of CD68 positive cells per hole in each VC obstruction-class. In both graphs, each data point represents a single explanted VC. Differences between groups were assessed using Kruskal–Wallis’s test with Dunn’s multiple comparisons test, P < 0.05 (*), P < 0.01 (**) and P < 0.0001 (****)

During analysis of CyQuant labels of the subgroup of 54 VCs, significantly more nuclei were observed in ‘VCs with Occlusions’ when compared with ‘VCs with Growths’ (p < 0.0001) and ‘VCs with Occlusions’ (p = 0.014, Fig. 4A). The difference in total cells was not significantly different between Empty VCs and VCs with Growths (p = 0.28).

Similarly, we quantified the number of CD68 positive cells in each hole of each catheter. A comparison of the mean number of CD68 positive cells between occluded EVDs and occluded VCs found that there was no significant difference in CD68 positive cells present (p = 0.7).

**Fig. 5:**
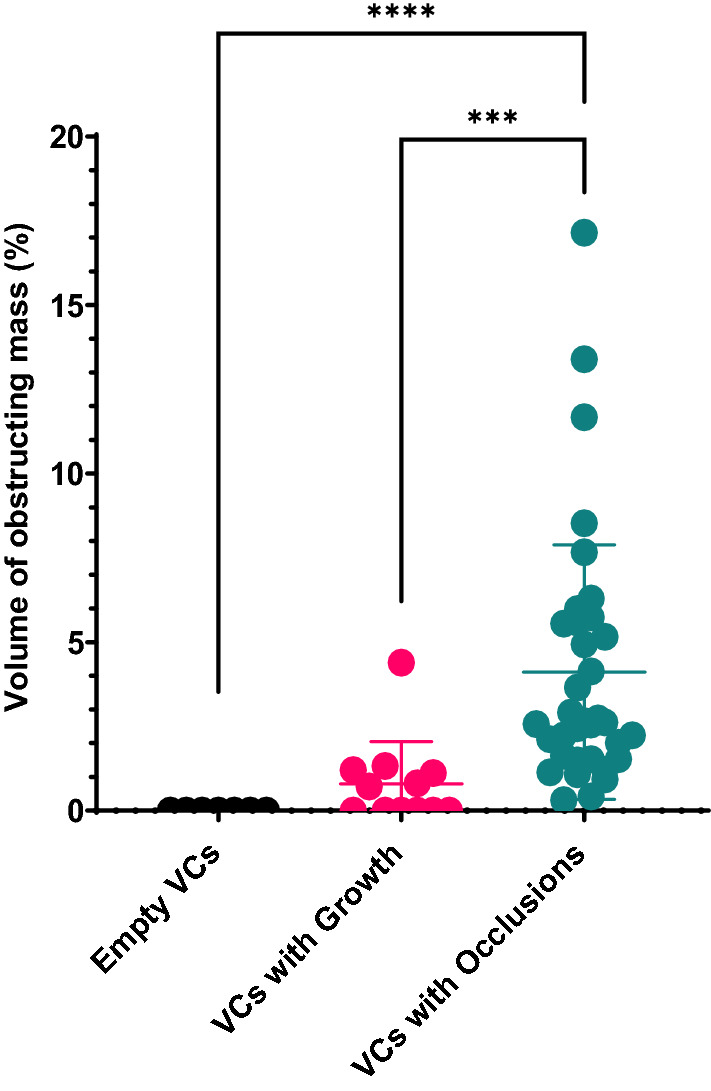
Percent obstruction of the total volume of the holes of each catheter. Each data point represents a value from a single explanted catheter. Differences between groups were assessed using Kruskal–Wallis’s test with Dunn’s multiple comparisons test, P < 0.001 (***), P < 0.0001 (****)

Next, we compared the total percentage of the catheter’s obstructed hole volumes between VC obstruction classes. Once again, we observed a significant difference between the Empty VCs and ‘VCs with Occlusions’ (p < 0.0001) and also between ‘VCs with Growths’ and ‘VCs with Occlusions’ (p = 0.0003, Fig. 5).

### Histopathological and immunohistochemical evaluation of tissue aggregates in explanted shunts

To get a qualitative understanding of the tissue features that were missed by the four IF labels and to confirm the presence of cell types observed with the confocal analysis, the 54 VCs were analyzed using traditional histology and IHC techniques. 72% (n = 39) of the VCs were observed to be invaded by tissue large enough to be excised and embedded for IHC processing. The common features observed are summarized below in Table 1.

**Table 1:** Tissues identified through histopathological evaluation of explanted VCs

Predominant tissue type	Sample size (%)
Choroid plexus	13 (24)
Vascularized glial tissue	13 (24)
Lymphocytic inflammation	16 (29)
Foreign body giant cell reactions	3 (5)

**Fig. 6:**
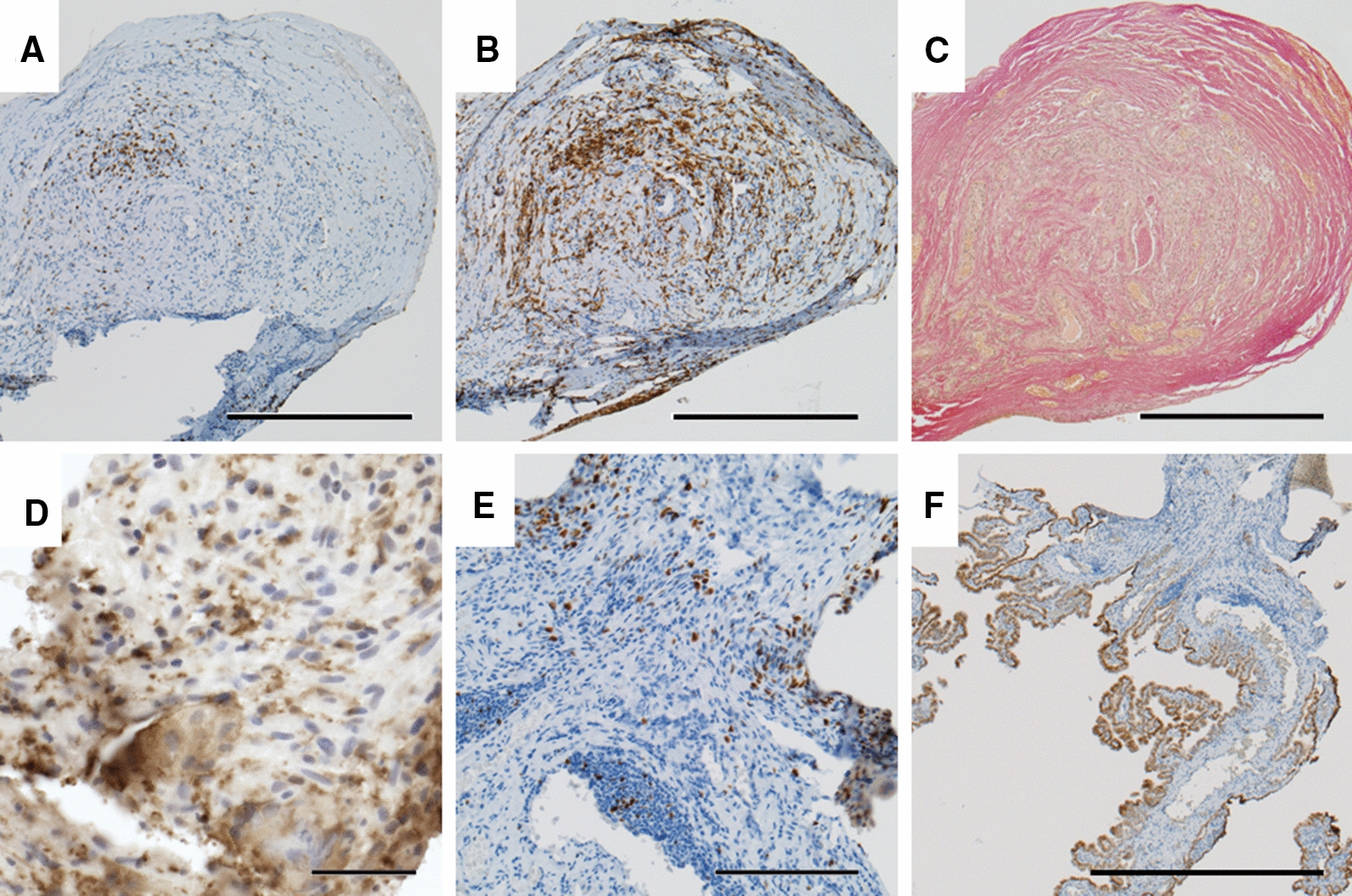
Representative sections of choroid plexus tissue plug extracted from a ventricular catheter (VC). Sections ‘a’ through ‘e’ are from the plug in the lumen whereas ‘d’ through ‘f’ are from the pedicles attached to the plug. **a** Population of T lymphocytes (stain for CD3; magnification, × 100; scale bar, 500 µm) (**b**) Populations of activated macrophages and microglia (stain for HLA-DR; magnification, × 100; scale bar, 500 µm) (**c**) Fibrosis (stain for collagen (pink); magnification, × 100; scale bar, 500 µm) (**d**) Activated microglia and macrophages (Iba-1 stain; magnification, × 400; scale bar, 50 µm) (**e**) Proliferating inflammatory cells (Ki67 stain; magnification, × 200; scale bar, 200 µm) (**f**) Cytoskeleton of choroid plexus epithelium (CK stain; magnification, × 40; scale bar, 1000 µm)

CP tissue in IHC stains matched our observations of morphological structures like the papillary structures observed in our confocal images that were suggestive of CP tissue. CP tissue was observed in 13 of the 54 VCs (24%, Fig. 6). In some cases, CP tissue appeared to be atrophic and in other instances showed intense chronic inflammatory reactions with proliferating lymphocytes and macrophages present (Fig. 6e).

Activated astroglial brain tissue was observed in 17 VCs (31%). In the 17 VCs with astroglial brain tissue, foreign body giant cell type reactions were frequently observed in tandem with microglial activation. (Additional file 1: Fig. S1). Less frequently we observed blood clots obstructing VC holes (Additional file 1: Fig. S2a). The IHC stains also revealed T lymphocytes, plasma cells, monocytes and macrophages at the interface of the tissue plug and the VC. (Additional file 1: Fig. S2c, d).

**Fig. 7:**
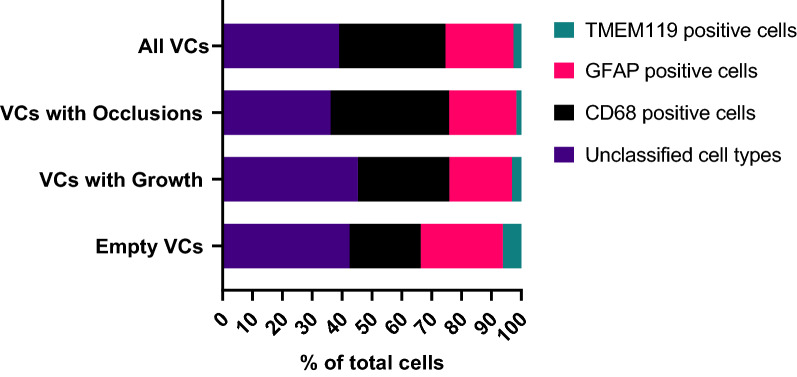
Percentage composition of each cell type forming the tissues obstructing VCs within each VC obstruction class

Across the 54 VCs that were analyzed using IF labels, an average of 70–75% of cells were identified and quantified (Fig. 7). IHC stains helped identify the remaining 25–30% of cells as being a mix of B and T lymphocytes, plasma cells, endothelial cells and foreign body giant cells.

### Linear mixed model analysis

Application of linear mixed models to understand associations between IF labelled cell types and clinical parameters revealed the following: implant duration was inversely related to the number of CyQuant positive nuclei (p = 0.043) and CD68 positive cells (p = 0.040). The number of revisions a patient had was found to be a significant predictor of TMEM119 (p = 0.005). 3-side 8-hole shunts were significantly different from 4-side 4-hole shunts in terms of GFAP positive cells (p = 0.03). However, there was no significant global effect for shunt type and GFAP positive cell count. The covariates namely age, gender, race, number of revisions, and reason for surgery, were not significant predictors of IF labelled cell types.

## Discussion

This study builds on previously published work [13, 14] and broadens the scope through quantification and a multicenter biobank. We developed and applied an objective, easily replicable image categorization scheme to 343 samples from our biobank. Using the degree of VC obstruction as a discretized measure we were able to normalize and compare VCs across patients. Unlike previous studies, our work relies on quantitative techniques to analyze tissue aggregates obstructing VCs and links the degree of VC obstruction to clinical parameters from the patients.

Clinical determination of VC obstructions did not always translate to a tissue aggregate obstructing the VC (Fig. 2B). Although the clinicians recorded the suspected cause of failure for 41.2% of explanted VCs as a “proximal obstruction” based on intraoperative assessment, we observed obstructive tissue aggregates on a majority of the VCs (61.5%, n = 211). Likewise, VCs that were clinically deemed to have failed by ‘causes other than proximal obstruction’ frequently contained obstructing tissue aggregates. One explanation for this inconsistency may be that VCs are sometimes recorded as obstructed by the surgeon if there is no flow through the VC during intraoperative shunt interrogation. Such a temporary stoppage of flow may occur if the VC shifts position over time, is not positioned optimally, or if tissue obstructing the catheter gets dislodged during explantation. Conversely, a patient may have a catheter with tissue aggregates obstructing it without flow being affected.

A significant difference between the VC obstruction categories was observed in the hydrocephalus etiology, myelomeningocele (Chi square p = 0.0426). Patients with myelomeningocele had a greater proportion of ‘VCs with Occlusions’ than other etiologies. Our data is consistent with the observation that patients with myelomeningocele exhibit the highest rate of failure of anti-siphon devices [25]. It has also been suggested that patients with myelomeningocele are predisposed to develop a sensitization to latex by exposure during surgery [26]. This sensitization could contribute to a systemic inflammatory response, which may be partially responsible for the greater proportion of VCs with Occlusions in patients with myelomeningocele. Perhaps the early exposure of their CNS and CSF system leaves them susceptible to stronger immune reactions to foreign bodies, such as VCs. Abnormal CSF contents within the first postoperative year, in patients with myelomeningocele, may also play a role [27]. Shunt-bound proteins have been shown to trigger immunological responses in some individuals [17].

EVDs were included in our analysis to give additional insight into how the response to catheters varied in the short term versus those implanted for longer periods of time. Additionally, the potential variances due to blood exposure were of interest to us. The cell types present in the tissue aggregates obstructing EVDs were not different from those obstructing VCs, suggesting that the early mechanisms driving obstruction are similar. In future work we will delve deeper into the differences between short- and long-term cellular responses by directly comparing EVDs and VCs.

In 41 of the 343 VCs, surgeons used intraluminal electrocautery (Bugbee wire monopolar electrocautery) in order to prevent bleeding and release the adherent tissue. Our analysis showed that 36 of these 41 VCs were classified as ‘VCs with Occlusions’, suggesting that intraluminal electrocautery did not disrupt tissue aggregates in all VC holes. Additionally, electrocautery did not account for the discrepancy between clinical determination of VC obstruction and the presence of tissue aggregates on VCs.

90 of the 211 ‘VCs with Occlusions’ were observed to have obstructive tissue lodged in holes closest to the VC insertion point into the ventricles (data not shown). This supports the hypothesis that some of the VC holes near the insertion point might be positioned outside of the ventricular space, close to the ventricular wall and brain parenchyma, increasing the risk of obstruction [8]. 35 of the 211 ‘VCs with Occlusions’ had obstructions exclusively in holes close to the proximal tip of the VC. Suboptimal VC positioning or VC shift during ventricular decompression may allow the tip to make contact with the septum pellucidum and position the proximal holes to be in contact with the CP [28]. This would presumably put the holes closer to the tip of the VC at higher risk of obstruction.

Our histopathologic analysis demonstrated that all the obstructed VCs showed varying degrees of inflammatory response, affirming previous findings [13, 14]. We attempted to differentiate between macrophages and microglia using the recently published microglia-specific marker from the Barres group [29]. TMEM119 positive / CD68 positive cells (microglia) were far fewer in number in invading tissue (1–6% of total cells) than TMEM119 negative / CD68 positive cells (cells of the monocyte/macrophage lineage, 22–27% of total cells), suggesting a smaller role of microglia than previously thought. This is in line with a recent report on the cells involved in neuroinflammatory responses to electrodes placed in the brain [30]. Macrophages and astrocytes have been reported to cause reactive astrogliosis and proliferation of glia at the site of injury [31, 32]. In future work we plan to look closely at the interplay between these two cell types.

Choroid plexus tissue was observed in 13 of 54 samples (24%), suggesting low overall involvement in VC obstruction. Early reports attributed a significant proportion of VC obstructions to CP tissue [33] while our data supports more recent reports of CP tissue being less common than and secondary to glial, connective tissue or chronic inflammatory responses [8, 13–15, 34, 35]. In our samples, CP tissue typically appeared as a large tissue aggregate protruding from holes showing clear signs of being avulsed from the ventricle.

Importantly, ependymal tissue was not observed in any of the obstructed VCs, which is in line with previous observations [8]. This does not however discount the ventricular wall as a source of cellular ingrowth. Ependyma-covered glial evaginations were observed by Del Bigio et al. to invade VC holes beginning one-week post-implantation. The peak of these evaginations were denuded, exposing underlying periventricular astrocytes [9, 36]. This description of evaginations matches the tissue aggregates observed in some of our VCs, which suggests the occurrence of a chronic inflammatory process initiated by contact and friction and/or suction of the ependymal wall against the VC. This could be exacerbated by the invasion of the ventricular wall by astrocytes and blood-derived macrophages, a notion supported by observations of ventricular zone disruption by astrocytes and macrophages as early as 24 h after blood exposure following intraventricular hemorrhage [37].

We observed an inverse correlation between CyQuant positive nuclei and duration of catheter implantation. One interpretation could be that longer lasting VCs are in a relatively stable environment with a dampened foreign body response as compared to VCs that lose patency after a short duration. Alternatively, VCs that get obstructed within a short duration are positioned such that there is more tissue contact than longer lasting VCs. This is a relationship that merits further investigation.

It is important for us to mention here the caveats of our methods of analysis and data collection. (1) The volume and appearance of tissue aggregates observed on the VCs may be an artifact of how the tissue breaks when the shunt is removed. (2) Whether tissue growths in the VC holes caused obstruction of the lumen was not explored. Future work will elaborate on this relationship. (3) In our IHC analysis some multinucleated foreign body giant cells also stained positive for TMEM119, suggesting that TMEM119 may not be specific to only microglia. Additionally, TMEM119, CD68, HLA-DR and Iba1 have been shown to identify different activation stages of microglia [38, 39]. Therefore, presentation of one antigen alone does not suggest that the microglia is activated, and likewise the absence does not exclude the activation. To capture the entire population of microglia we stained for all 4 antigens. (4) Subtle genetic differences dependent on jurisdiction, socio-economic status and nutritional differences that may predispose patients to inflammatory responses were not investigated in this work but have previously been discussed [7].

Additionally, the effect of bleeding on insertion, the speed of shunt insertion, and barium sulfate or antibiotic impregnation of the silicone catheter, were not considered during our analysis. In the future we address the impact of these confounding factors and direct attention towards mechanistic relationships involving flow, VC materials and design, implant duration, number of revisions, and infections.

## Conclusion

In this study, we characterized the tissue aggregates obstructing ventricular catheters and sought to link degree of obstruction with clinical parameters. Our data show that the majority of catheters have obstructing tissue aggregates. Additionally, there is a discrepancy between the surgeon’s clinical assessment of catheter failure and the observed degree of obstruction. Our data suggest that the flow rate through a catheter hole acts in conjunction with other factors and does not independently influence the degree of obstruction. Importantly, we demonstrate that varying degrees of inflammatory responses are involved in obstructing catheters, with astrocytes and macrophages, not microglia, being the most abundant contributors. Future studies should parse the contribution of tissue-contact caused by over-drainage or suboptimal placement of the catheter and foreign body response to the VC mediated by astrocytes and macrophages. Ventricular catheter obstruction is likely a multifactorial failure, and any strategy to effectively mitigate catheter obstruction should consider multiple mechanisms of obstruction.

## Supplementary Information

**Additional file 1:** **Table S1.** Primary antibodies used for immunohistochemistry. **Table S2.** Summary of patient demographics, hydrocephalus etiologies, suspected causes of shunt malfunction in population of 265 patients. **Figure S1**. Representative sections of astroglial tissue plug extracted from a VC. **Figure S2.** Representative sections of tissue aggregates extracted from different VCs.

## Data Availability

The raw datasets analyzed during the current study are not publicly available due to privacy concerns surrounding HIPAA but the deidentified data and the processed data required to reproduce these findings are available from Dr. Carolyn Harris, Ph.D., Wayne State University, Dept. of Chemical Engineering and Materials Science, 6135 Woodward Avenue, Detroit, MI 48,202. Email: caharris@wayne.edu.

## Abbreviations

CSF: Cerebrospinal fluid
CP: Choroid plexus
CNS: Central nervous system
ETV: Endoscopic third ventriculostomy
EVD: External ventricular drains
VC: Ventricular Catheter
VP: Ventriculoperitoneal
IF: Immunofluorescent
IHC: Immunohistochemistry
SD: Standard deviation
