# Supplementary material for: A multicenter retrospective study of heterogeneous tissue aggregates obstructing ventricular catheters explanted from patients with hydrocephalus
Source: Fluids Barriers CNS. 2021 Jul 21;18:33. doi: 10.1186/s12987-021-00262-3 (PMC8293524; doi:10.1186/s12987-021-00262-3)
Supplement: Supplementary file 1 — Additional file 1: Table S1. Primary antibodies used for immunohistochemistry. Table S2. Summary of patient demographics, hydrocephalus etiologies, suspected causes of shunt malfunction in population of 265 patients. Figure S1. Representative sections of astroglial tissue plug extracted from a VC. Figure S2. Representative sections of tissue aggregates extracted from different VCs. [file 12987_2021_262_MOESM1_ESM.docx]

**Electronic Supplementary Material:**

**Title:** Examination of the Heterogeneous Foreign Body Response in Obstructed Ventricular Catheters Implanted in Patients with Hydrocephalus

**Supplementary Table 1. Primary antibodies used for immunohistochemistry**

| **Primary antibody target** | **Description; commercial source** | **Use** |
| --- | --- | --- |
| AIF-1 (allograft inflammatory factor-1 / Iba1) | Rabbit polyclonal; Synaptic Systems 234003 (footnote 1) | Microglia / macrophages |
| CD138 (syndecan 1 / SDC1) | Clone B-A38 mouse monoclonal; Cellmarque 138M-16 | Plasma cells |
| CD20 | Clone L26 mouse monoclonal; Dako M0755 | B lymphocytes |
| CD3 | Rabbit polyclonal; Dako A0452 | T lymphocytes |
| CD34 | Clone QBEND10 mouse monoclonal; Dako M7165 | Endothelial cells |
| CD4 | Clone 4B12 mouse monoclonal; Dako M7310 | ‘helper’ T lymphocytes |
| CD68 (lysosomal LAMP4) | Clone PG-M1 mouse monoclonal; Dako M0876 | Macrophages / microglia |
| CD8 | Clone 4B11 mouse monoclonal; Novocastra NCL-L-CD8-295 | ‘cytotoxic’ T lymphocytes |
| CK AE1AE3 (cytokeratins 1-8,10,14-16,19) | mouse monoclonal cocktail; Dako M3515 | Choroid plexus epithelial cells |
| Collagen IV (alpha 1 / COL4A1) | Clone CIV22 mouse monoclonal; Dako M0785 | Vascular basement membrane |
| Glial fibrillary acidic protein (GFAP) | Rabbit polyclonal; Dako Z0334 | Astrocytes |
| HLA-DR (major histocompatibility complex, class II, DR) | Clone CR3/43 mouse monoclonal; Dako M0775 | B lymphocytes, monocytes, activated microglia and T lymphocytes |
| Ki67 | Clone MIB-1 mouse monoclonal; Dako M7240 | Proliferating cells |
| Myeloperoxidase (MPO) | Rabbit polyclonal; Dako A0398 | Neutrophils |
| NeuN (RNA binding protein fox-1 homolog / RBFOX3) | Clone A60 mouse monoclonal; Millipore MAB377 | Neurons |
| Neurofilament (light / heavy) | Clone 2F11 mouse monoclonal; Dako M0762 | Axons |
| TMEM119 (C terminal) | Rabbit polyclonal; Abcam 185333 (footnote 1) | Microglia |

Footnote 1 - immunostaining performed manually with biotinylated secondary followed by streptavidin peroxidase and diaminobenzidine detection

**Table 2: Summary of patient demographics, hydrocephalus etiologies, suspected causes of shunt malfunction in population of 265 patients**

| **Variable** | **No. of samples (%) imaged using brightfield microscopy** | **No. of samples (%) imaged using confocal microscopy and sectioned for histology** |
| --- | --- | --- |
| **Sex** | | |
| Male | 198 (57.77) | 35 (64.8) |
| Female | 144 (41.98) | 19 (35.2) |
| **Patient age at surgery** | | |
| 0 to 2 | 86 (25.07) | 12 (22.2) |
| 3 to 5 | 57 (16.61) | 15 (22.78) |
| 6 to 13 | 108 (31.49) | 15 (22.78) |
| 14 to 18 | 56 (16.33) | 5 (9.25) |
| 19 to 33 | 27 (7.87) | 3 (5.56) |
| 34 to 48 | 9 (2.62) | 4 (7.4) |
| **Etiology of hydrocephalus** | | |
| Myelomeningocele | 45 (13.12) | 2 (3.7) |
| Brain tumor (all types) | 21 (6.12) | 4 (7.4) |
| Intraventricular hemorrhage of prematurity | 149 (43.4) | 31 (57.4) |
| Congenital |  |  |
| Aqueductal stenosis | 27 (7.87) | 4 (7.4) |
| Dandy Walker malformation/obstructive arachnoid cyst | 9 (2.6) | 1 (1.8) |
| Congenital – not specified | 28 (8.16) | 7 (12.96) |
| Macrocephaly/ventriculomegaly without apparent CNS structural abnormality | 7 (2.04) | 4 (7.4) |
| Others |  |  |
| Craniosynostosis | 2 (0.5) | - |
| Postnatal meningitis | 11 (3.2) | - |
| Trauma | 7 (2.33) | - |
| Not specified | 37 (10.78) | 1 (1.8) |
| **Reasons for shunt revision:** | | |
| Disconnection of shunt | 14 (4.08) | 4 (7.4) |
| Fractured shunt | 3 (0.8) | 1 (1.8) |
| Obstruction, distal | 12 (3.5) | - |
| Obstruction, proximal | 250 (72.8) | 31 (57.4) |
| Obstruction, valve | 21 (6.1) | - |
| Externalization for suspected infection | 42 (12.24) | 11 (20.37) |
| EVD removal and conversion to VP shunt | 28 (8.16) | 9 (16.67) |
| Shunt failure at unspecified location | 4 (1.16) | 1 (1.8) |


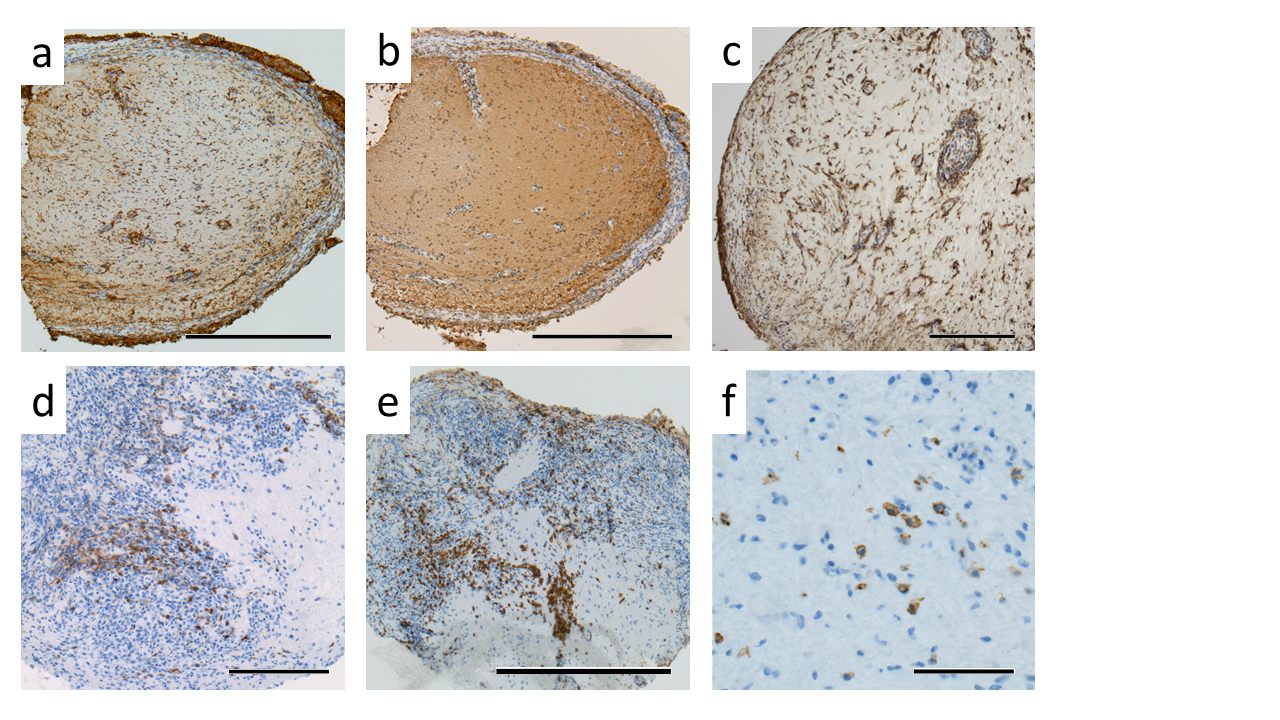


Supplementary Figure 1: Representative sections of astroglial tissue plug extracted from a VC. (a) Microglia and macrophages in brain tissue and foreign body giant cells at the interface (stain for HLA-DR; magnification, ×100; scale bar, 500 µm) (b) Dense glial core (GFAP stain; magnification, ×100; scale bar, 500 µm) (c) Microglia in brain tissue and giant cells at the interface (stain for Iba-1; magnification, ×200; scale bar, 200 µm) (d) Inflammatory B lymphocytes (CD-20 stain; magnification, ×400; scale bar, 200 µm) (e) Inflammatory T lymphocytes (CD-3 stain; magnification, ×100; scale bar, 500 µm) (f) Rare plasma cells (CD-138 stain; magnification, ×400; scale bar, 100 µm)


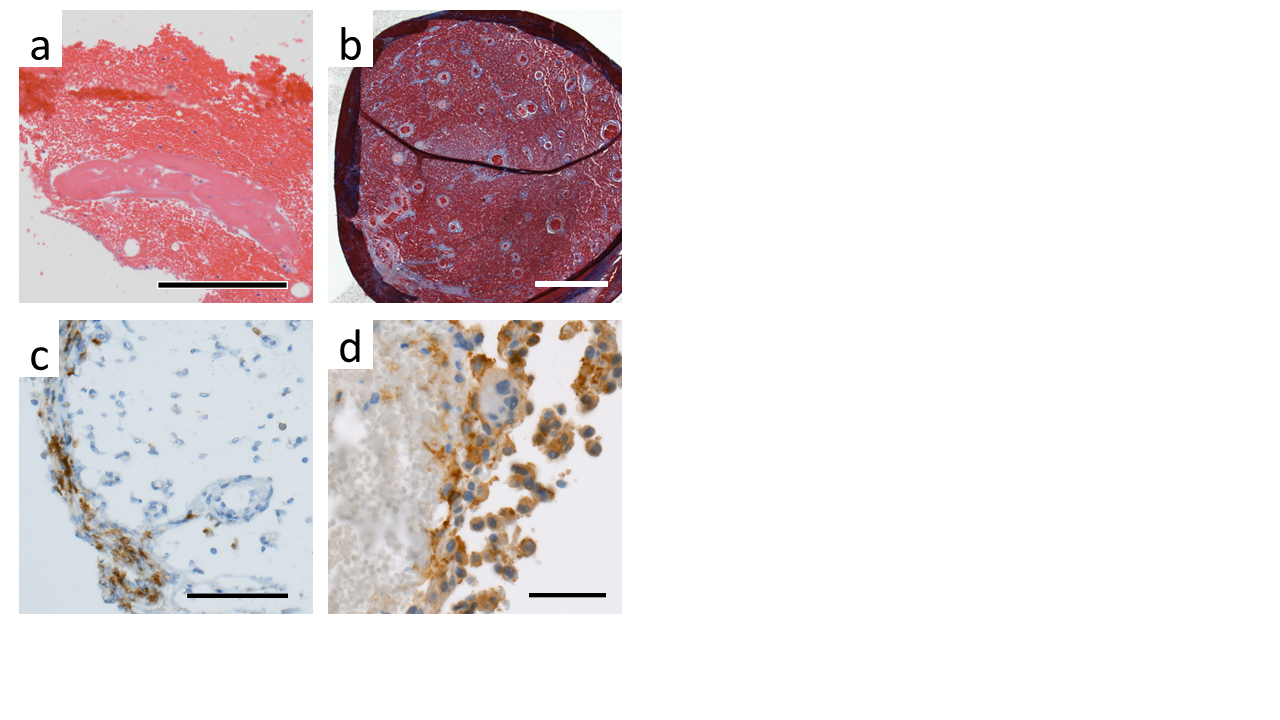


Supplementary Figure 2: Representative sections of tissue aggregates extracted from different VCs. (a) Cells in a blood clot plugging a VC hole (H&E stain; magnification, ×200; scale bar, 200 µm) (b) Fibrotic blood vessels in a highly vascularized brain tissue plug (Masson stain; magnification, ×100; scale bar, 200 µm) (c) T lymphocytes in tissue plug concentrated at interface with catheter (CD-3 stain; magnification, ×400; scale bar, 100 µm) (d) Plasma cells, monocytes and macrophages at the edge of tissue plug (MPO stain; magnification, ×600; scale bar, 50 µm)
